# Translating community voices to build meaningful engagement: a Community Health Worker led study

**DOI:** 10.3389/fpubh.2026.1728914

**Published:** 2026-02-26

**Authors:** Lauren M. Workman, Julie Smithwick, Kimberly C. Rawlinson, Mark M. Macauda, LaShandra Morgan, Whitney Davis

**Affiliations:** 1 Center for Applied Research and Evaluation, Arnold School of Public Health, University of South Carolina, Columbia, SC, United States; 2 Department of Health Services Policy and Management, Arnold School of Public Health, University of South Carolina, Columbia, SC, United States; 3 Center for Community Health Alignment, Arnold School of Public Health, University of South Carolina, Columbia, SC, United States; 4 Colorectal Cancer Prevention Network, University of South Carolina, Columbia, SC, United States; 5 Breathe Easy SC, Charleston, SC, United States; 6 Darlington County First Steps, Lamar, SC, United States

**Keywords:** community engagement, community health, Community Health Workers, health equity, qualitative research

## Abstract

**Background:**

Understanding community perspectives on barriers and facilitators of meaningful engagement, equitable decision making, and sustainable community improvement is critical for advancing health equity. Community Health Workers can make significant contributions to research teams, as they are bridges into communities and bring unique perspectives. This study describes community voices on barriers to authentic engagement and insights on strategies to build trust, as well as the way in which these data were translated into a tangible assessment tool and a community-facilitated learning academy.

**Methods:**

A team of Community Health Workers led a series of 24 ‘open mic’ discussions in four South Carolina communities. Verbatim transcripts were analyzed inductively.

**Results:**

Participants were mostly female (83%), African American (88%), and had a range of education, ages, and levels of engagement. Community concerns (violence, lack of resources, and insufficient economic development) were a primary focus of conversation. Barriers to community engagement include not having sufficient input into what the engagement focus should be, mistrust in outsiders (which in part stems from a history of racism), lack of voice in decision-making, and broken promises. Participants view trust as the essential building block for community engagement, which takes genuine connections, listening, consistency, commitment, and follow-through. These data were used to inform the Prioritizing Long-lasting Actionable Community Engagement & Equity (P.L.A.C.E.) Academy, which aims to equip community leaders with tools for meaningful engagement.

**Discussion:**

Our findings confirm the Aligning Systems for Health framework, which calls for attention to equity, power dynamics, trust, and the need to let community generate priorities and solutions. Academic, public health, healthcare and social service partners must work with communities to ensure that community issues are addressed respectfully and equitably. Our data driven curriculum is a critical step toward stronger engagement, enhanced communication, and improved health outcomes. Developing research teams that include Community Health Workers is a pragmatic strategy that can lead to better insights, greater trust, balances in power, and ultimately, greater health equity.

## 1 Introduction

The field of public health recognizes the influence of community and structural factors as key drivers of health and wellbeing (1). Thus, shifting policies, systems and community contexts are key health promotion levers (2). A key strategy to operating policy, systems, and community levers for change is the application of participatory and community engaged approaches, which provide opportunities for collaboration, shared power and decision making, and the acknowledgement that no single factor influences health (3). Community engagement, as defined by the World Health Organization, is “a process of developing relationships that enable stakeholders to work together to address health-related issues and promote well-being to achieve positive health impact and outcomes” (4). While robust evidence documents the positive relationship between community engagement and health outcomes (including health equity), promoting community engagement is difficult. This is a particular challenge in communities who have experienced past mistreatment and resulting mistrust (5). While the concept of community engagement in research is well accepted, the most effective strategies to promote it are still being studied (6). It is also accepted that community engagement can be considered as a continuum, ranging from interactions with limited, episodic involvement to those that build long term, sustainable partnerships and shared leadership (7). Evidence suggests that moving along the continuum to shift more power into communities and build more trust and transparency can have transformational and sustainable effects in communities (8). Thus, more efforts and strategies are needed to build meaningful community engagement and shared decision making. However, prior research indicates that communities may require intentional support and training to build skills and capacity for engagement (9, 10). Moreover, the delivery of this support and training through structured opportunities for communities is recommended and is shown to promote increased community engagement (11, 12).

Here, meaningful community engagement can be defined as a process that allows communities to collaborate on a deeper level for shared decision making, rather than occasional consultation or superficial interactions (6, 13). Shared, or equitable, decision making considers the ways in which power is distributed between researchers and communities, but more guidance in the literature is needed to guide this process in community based research (14, 15). Thus, to explore strategies to promote meaningful community engagement and equitable decision making for sustainable community health improvement, we received funding through Robert Wood Johnson Foundation’s Aligning Systems for Health initiative (16). Their Aligning Systems for Health framework recognizes the importance of community voices, but also the need to identify and use the most effective strategies to engage those voices (17, 18).

Regardless of discipline, there is consensus that understanding the lived experience of individuals most affected by health conditions, social factors, or other inequities is critical to understanding root causes that affect health and wellbeing (19, 20). One strategy to increase community engagement is having Community Health Workers (CHWs) lead implementation efforts (21, 22). The American Public Health Association defines a CHW as someone who is “*a trusted member of and/or has an unusually close understanding of the community served. This trusting relationship enables the worker to serve as a liaison/link/intermediary between health/social services and the community to facilitate access to services and improve the quality and cultural competence of service delivery*” (23). CHWs serve as advocates and support the increased capacity of communities, including participating in research and evaluation (24). As trusted persons in the community, CHWs can serve as conduits between researchers and community, allowing for greater insights and information into community contexts and realities (25). Including CHWs on research teams can also facilitate reach and engagement of communities, including underserved populations, in ways that traditional research approaches may not (25, 26). Additionally, the involvement of CHWs in the research process can enhance shared decision making by creating an environment that allows the views of the community to emerge (27, 28).

The purpose of this paper is twofold: (1) to describe our research partnership with CHWs to gather residents’ perspectives on barriers to community engagement, and their insights on strategies to build trust and (2) to illustrate how we translated this data into a CHW led pilot program called the Prioritizing Long-lasting Actionable Community Engagement & Equity (P.L.A.C.E.) Academy, which aimed to build community capacity for engagement among community leaders.

## 2 Methods

The University of South Carolina Institutional Review Board approved this study.

### 2.1 Setting and partners

This study was conducted in four counties in South Carolina with diverse characteristics, including two rural and two urban, representing four different regions of the state (Table 1). Organizations in the counties were invited to partner with our study team based on recommendations from state and community leaders and their local infrastructure to conduct community engaged research. We sought communities with an active organization or group to lead local efforts, that had been working in their community for at least 2 years, and the ability to find a CHW in the area. Additional criteria included variation in community setting (e.g., urban and rural contexts), variation in topic area and/or population served, willingness to share lessons learned with the research team, and current representation from each sector involved in the initiative. Using these criteria, we identified four partner organizations that reflected variation across these dimensions. After identification, we contacted each organization to assess interest in partnering and conducted follow-up discussions to confirm alignment with project goals and overall fit. Three of the four organizations agreed to participate, which included a county coordinating council, a youth empowerment initiative, a health coalition, and an informal group of community partners organized for community wide impact. In the fourth community, a group of individuals who were already working together served in an advisory capacity for their community. These organizations and groups were critical to understanding the communities and gaining access. For example, community organizations facilitated connections between the research team and the community, brokered connections to identify CHWs to participate on the research team, and provided concrete supports (including meeting space), and supported the dissemination of results and findings into the local community.

**Table 1:** Selected county health rankings community indicators (41).

Community	Community partner description	Population living in a census-defined rural area	Individuals who do not identify as non-Hispanic White	Children (<18) in poverty	Poor or fair health	Population with access to exercise opportunities	Adults reporting that they always, usually or sometimes feel lonely
1	A county coordinating council	57.1%	45.2%	30%	20%	46%	34%
2	A health coalition	65.5%	65.2%	29%	23%	49%	36%
3	A youth empowerment organization	8.6%	58.3%	20%	17%	74%	31%
4	An informal group of community leaders	29.5%	32.5%	20%	16%	72%	32%

### 2.2 CHWs as research partners

A team of two CHWs from the Center for Community Health Alignment (CCHA) and two academic public health researchers developed the initial research plan and goals. For implementation, three more CHWs that currently lived in (and were native of) three of the counties were hired as Community Health Worker Researchers (CHWRs); the fourth community was led by a CCHA CHW who was native to that community. CHWRs were recruited via widely shared job postings in partnership with local organizations. Once hired, formative interviews were conducted with the CHWRs to understand their research experience and skills, and to determine their research training needs. They were then provided with tailored training by study team members with methodological expertise in qualitative methods to ensure all CHWRs were comfortable using them. The CHWRs also received certification in the ethical conduct of research.

### 2.3 Recruitment

Once CHWRs were trained, we hosted community discussion sessions in the four counties. The sessions were called “open mic” (microphone) community conversations.^1^ Several methods were used to recruit, including direct recruitment by CHWs and project partners, word of mouth, flyers, and social media postings. Some participants invited friends to attend with them or to participate in the next cohort (snowball sampling). Sessions were held in diverse neighborhoods; individuals from different demographic groups were recruited to ensure a range of perspectives. A total of 24 sessions were held across the four communities, ranging from 5 to 14 participants. Table 2 provides participant demographics by community. Participants were mostly female and African American, but were diverse in education, age, and current level of community engagement.

**Table 2:** Participant demographics.

Characteristic	Community 1 *N* = 15	Community 2 *N* = 17	Community 3 *N* = 23	Community 4 *N* = 16	Total *N* = 71
Race					
Black or African American	13 (86.7%)	16 (94.1%)	17 (79.3%)	16 (100%)	62 (87.3%)
White	1 (6.7%)	1 (5.9%)	3 (13.0%)	0 (0%)	5 (7.0%)
Other	1 (6.7%)	0 (0%)	3 (13.0%)	0 (0%)	4 (5.6%)
Hispanic/Latino?					
Yes	1 (6.7%)	0 (0%)	0 (0%)	0 (0%)	1 (1.4%)
No	14 (93.3%)	16 (100%)	22 (100%)	16 (100%)	68 (98.6%)
Education level					
Some high school, high school diploma, or GED	3 (20.0%)	2 (11.8%)	6 (26.1%)	1 (6.2%)	12 (16.9%)
Some or completed technical/ trade school degree	2 (13.3%)	6 (35.3%)	2 (8.7%)	3 (18.8%)	13 (18.3%)
Some or completed college degree	10 (66.7%)	9 (52.9%)	15 (65.2%)	12 (75.0%)	46 (64.8%)
Gender					
Male	2 (13.3%)	4 (23.5%)	5 (21.7%)	1 (6.3%)	12 (16.9%)
Female	13 (86.7%)	13 (76.5%)	18 (78.3%)	15 (93.8%)	59 (83.1%)
Age					
18–24	2 (13.3%)	0 (0%)	0 (0%)	0 (0%)	2 (2.9%)
25–44	9 (60.0%)	4 (23.5%)	10 (43.5%)	6 (37.5%)	29 (40.8%)
45–64	4 (26.7%)	5 (29.4%)	11 (47.8%)	9 (56.3%)	29 (40.8%)
65–74	0 (0%)	8 (47.1%)	2 (8.7%)	1 (6.2%)	11 (15.5%)
In your opinion, how involved are you in things like community action groups, community coalitions, and/or nonprofits working in your community?					
Not involved at all	6 (40.0%)	2 (11.8%)	2 (8.7%)	1 (6.3%)	11 (15.5%)
I have some involvement	4 (26.7%)	7 (41.2%)	4 (17.4%)	3 (18.8%)	18 (25.4%)
Fairly involved	2 (13.3%)	3 (17.6%)	4 (17.4%)	2 (12.5%)	11 (15.5%)
Very involved	2 (13.3%)	4 (23.5%)	5 (21.7%)	1 (6.3%)	12 (16.9%)
I have a leadership role	1 (6.7%)	1 (5.9%)	8 (34.8%)	9 (56.3%)	19 (26.8%)

### 2.4 Session format

We hosted two series of three open mic sessions in each of the four communities (24 sessions). Participants were asked to attend all three sessions in the series since conversations were designed to build across sessions. They received a $30 gift card for each session attended, as well as a meal for in-person sessions. While the COVID pandemic required our team to shift between virtual and in person sessions, we used a structured protocol for all sessions to ensure consistency.

Sessions were facilitated by a CHWR, conducted in English, and lasted about 1.5 h. The first two sessions in the series were focused on learning about the community, hearing members’ perspectives on effective strategies for community engagement, facilitators of and barriers to engagement, and key community challenges. A discussion guide was used for both sessions, but session two was designed to build upon the previous conversation. Session three was for member checking whereby participants received a summary of sessions one and two and then provided additional feedback. Discussions were recorded with permission and the recordings were transcribed for analysis.

### 2.5 Analysis

Analysis began as data were collected, with discussions among all research team members about early findings and interpretations. Formal coding of transcripts used Dedoose, a qualitative data analysis software (29). The research team, including both public health researchers and CHWs from CCHA and CHWRs, analyzed all transcripts using an inductive approach. Such approaches allow for concepts or themes to emerge from raw data, rather than using prior assumptions or theory to guide the analysis (30). We used the constant comparison technique, with repeated review of data to allow key themes to emerge (31). These themes are presented in the results section. We used member checking in the last session to verify initial interpretations and to improve the credibility of our findings; feedback gathered helped clarify important nuances about the way that results were portrayed and gather additional details (for example, breaking down acronyms to ensure clear understanding) (32).

## 3 Results

Participants shared varied perspectives in the open mic conversations. These perspectives emerged in themes related to community assets, barriers to community engagement, and the needs and conditions required to facilitate more authentic engagement. While the focus of this paper is to describe participant’s perspectives on barriers to community engagement and their insights on strategies to build trust, we include perceived assets below as not to contribute to a community deficit paradigm.

### 3.1 Sense of connection and pride are key community assets

In defining community, participants focused on relationships and connections rather than places. For example, a participant from Community 3 explained, “*community to me is more than the place where I live…my community…it’s just people that I can help*.” Many reflected pride in their communities, it was ‘home’ to them. They shared stories of growing up in these communities, setting down ‘roots’, and having a connection to the people they have shared experiences with. Overall, participants said their communities have strong, resilient roots and people look out for one another. They noted that their communities include people who share common goals. For example, someone from Community 1 shared their perspective of how people are a community asset: “*There are a lot of really, really good people here…and a lot of people who just want what’s best for their children no matter which socioeconomic group they are a member of.*”

The strong, caring spirit of the communities was evident in conversations, as was the desire to help their communities thrive. Though much of their conversations focused on community issues, it was clear that participants felt their communities were caring places and they were optimistic about the future. For example, a participant from Community 4 shared their views on how people from their area come together in times of need:

*“Some of the good qualities of [our community] is…sometimes the things that people don't see…meaning when tragic things happen, people from all walks of life network together to make sure people are okay. So, the connection…that we can band together. And the younger generations are doing the same thing…I'm amazed by some of the younger generations.”*

### 3.2 Failure to acknowledge local history can be a barrier

One theme was the history of racism and its connection to many of the social issues communities face. Two communities shared specific examples in their history of traumatic events rooted in racism. These events are an unacknowledged ‘elephant in the room’ that must be addressed and reconciled for true healing and progress. Another theme was related to the history of African American communities being ignored. For example, a participant from Community 3 explained how history shapes the perception of their community:

*“[our community] has a lot of history behind it. There are a lot of people who don’t necessarily talk about…but, currently, [our community] has a very negative connotation attached to it because of the violence and just the negative mindsets.”*

There was also discussion about how information needed to access resources and events is kept from certain populations, including people of color. Therefore, a need was identified to diversify methods of communication with the community to ensure information reaches those who need it most. A participant from Community 4 shared their perspective on this:

*“I know that there are resources there, but the disconnect in the community is that everybody doesn't know where these resources are, or they don't know how to communicate. And then when you go to ask for whatever resources, instead of being assisted in the process of identifying how you can get help, it is belittling to people that do not really know what they need to ask. Customer service is a huge thing that we need to work on around here because you don't always get people that have ways to meet people where they are.”*

Participants frequently discussed a history of broken promises from local policymakers, developers, and others, which leads to an overwhelming lack of trust in elected officials and other decision makers. Participants explained that politicians come into their communities to campaign, making promises to get votes, but are never seen again once elected. Someone from Community 2 explained:

*“[They] want us to come out and vote or come to their meetings. It's like once they're where they need to be they disappear. And the thing is they'll come out, they'll shake your hand. ‘You need anything? I got somebody in my office that works with that.’ You reach out and email them and they don't even contact you back.”*

Others shared that efforts to engage the community thus far have been selective, at the convenience of outsiders. Moreover, these selective engagement opportunities are seen as inauthentic or disingenuous; for example, to create a photo opportunity or to raise a politician’s profile. For example, a participant from Community 1 described it as: “*doing the least amount to look good*.” Another participant from Community 2 echoed these frustrations, explaining:

*“It seems like around the time it's time to vote for people, you see all these people in the communities, taking pictures and all these things, and then once you've been elected…you don't see these people. It's like where y'all gone? Because I live in a community that that has happened. And it's like, ‘What have you done for the community?’ So, then when the smoke is clear…you don't see these politicians out here advocating for these communities at all.”*

### 3.3 Concern over critical social issues creates a barrier

Conversations often tracked back to community social issues that affect quality of life and how these issues are oftentimes top of mind for community members, whereas sometimes those that come into their communities to work with them have their own priorities and may not understand or may ignore these issues. Many participants voiced concerns over violence and how it hinders sense of community and safety. Some explained that they have been accustomed to violence, drugs, crime, and other destructive behaviors, but, as someone from community 3 explained, “*we do not have no voice at all*.”

One participant from Community 2 explained that they came to the open mic discussion specifically to voice their concerns over violence in their community:

*“The major problem that I see…is the violence. There's been a lot of shootouts there [in our apartment parking lot]. Matter of fact, our building just got shot up. I know this probably don't have anything to do [with this] because I did read over the [recruitment flyer] far as the economic, the food, and the health, and stuff. But this is just a concern that I'm having…and I’m in contact with a lot of people. And it's like nobody's caring. There's been murders out there, and see, this is just a major concern.”*

Participants expressed how there is good happening in their communities, but it is overshadowed by the violence and other disorder that commonly gets reported in the news and media. People commented on how other aspects of neighborhood disorder including crime, substance abuse, drug dealing, gambling, and gang activity impact their quality of life too. One resident from Community 2 explained how gang activity impacts their community:

*“I know that our community is [name of gang]. They marked that territory months ago. This is the first time of me living out there for five or six years that I've ever seen it to where they're marking their territory…now our kids are exposed to that type of conduct.”*

Others commented on the lack of pride some have in their neighborhood, which contributes to a sense of apathy. Participants talked about how in the past, residents used to sweep their stoops and participate in other community activities. Because of the many social issues in their communities, participants shared that there is a sense of hopelessness amongst some residents. The lack of action, development, and growth in their communities can prevent people from getting involved. One participant from Community 4 shared how people can feel hopeless: “*Sometimes you been beat down so bad by the system and people…you are just like, what’s the use?”*

The lack of engagement with teens and young adults was a barrier. A participant from Community 1 explained their concerns about youth: “*I’d say the younger people are getting left behind…we actually forgot about the youth and now, they are looking to the others who are actually getting them in a lot of trouble.”* By listening to the ideas and desires of young people, they may become empowered to engage more. Participants discussed the need for programs and support to guide youth, as they can get involved in gangs and other bad influences if they do not have activities and mentoring in the community. Participants across the state emphasized the need for free and low-cost outlets for youth. One participant from Community 2 explained:

*“[I]n my building…those children migrate from the bottom to the top of that apartment complex because they're just looking for something to do. It's nothing for them to do. So, it's like, 'Okay, what can we do as a community to show these children that, 'Hey, we have your back. What do you need?' Because parents be doing their own thing. So, if there's no community engagement out there for the youth, their outlet is going to be drugs, alcohol, gang violence and dropouts in school and things like that. So, the youth is more important to me than anything.”*

Lack of affordable housing, economic development, and other resources (including education, childcare, transportation, and jobs) were also significant concerns. Residents discussed the lack of development in certain neighborhoods, and how development has displaced residents due to high costs or broken promises (e.g., promising local improvement or employment that never materializes). Participants explained the complexities of housing and how issues like gentrification, unfair housing subsidy rules, and lack of affordability affect lives. A participant from Community 4 shared:

*“Gentrification was the one of the worst things that has ever happened to us. They call it revitalization, but it's not. When you are taking from people that work hard every day and you are trying to mask it as revitalization, that is an ugly thing… On the north side [of town], they revitalized a whole lot, but when you take home ownership from people, that is not a good thing.”*

Transportation is lacking, especially for youth and senior citizens in rural communities. Two communities in particular have very rural areas. Residents from those counties spoke about how geographic isolation in rural communities result in lack of access to resources including jobs, education, childcare, quality grocery stores, and healthcare. Participants also spoke of how the lack of resources for youth and family recreation is a key challenge. Someone from Community 1 explained:

*“Once you leave school, there's nothing else to do. We do have the little parks that they've opened in town and but, there needs to be more community type things. The ballpark right now is the only place where they have any of that, but it's not cheap to sign up to play baseball…there's a lot of parents who just can't afford that. We've got to find other ways to reach out to these kids in this community, and let the parents know that where they're going is safe and that we've put an effort into it.”*

In addition, participants shared how the lack of resources in their small towns impacts their ability to keep young residents. For example, residents from one of the very rural communities explained how the lack of economic and infrastructure development in small towns results in young people leaving the community. A resident of Community 3 explained their perspective:

*“One of the challenges that we have is being a community that historically was a farming community, but that's changing. It left us in a place where we didn't get the infrastructure within the community and where industry…they wouldn't come because it wouldn't benefit them. That hurt us because our young people, they don't have the same opportunities other communities have. They go to [names of nearby cities]…they go to other places where there's more work.”*

### 3.4 Lack of opportunities for community voices in decision making

Participants shared frustration with outsiders coming into neighborhoods and making decisions without community input, which leaves people feeling disenfranchised. A participant from Community 1 explained:

*“It seems to play out where somebody tells you, ‘This is what we going to do. Whether you like it, don't like it.’ A lot of time people just come, especially [when] you’re talking about people with clout and power. Sometimes they'll pacify you and listen. Or pretend they're listening, but they already have their mind made up.”*

Another resident from Community 4 explained the need to ask residents for their perspectives in a respectful way:

*“When we went through urban renewal, the people who are sitting at the table--they should actually be from the community. It shouldn't be,’ we're going to tell you what we're going to do.’ It should be, ‘you tell us, or let's sit down and have a conversation to see what's needed’… you have to meet people where they are… and it might not necessarily be that professional setting. And we sit down and listen and not just discredit them because they're not as articulate or hadn't been to school for 30 years. If they have something to say, you should listen, not brush them off as they're not educated.”*

Participants in one county reported being left out of decisions on what schools will remain open or be consolidated, or what incentives businesses will receive for moving into an area. Others voiced frustration over liquor stores, payday loan companies, and other negative businesses preying on poor communities and local government showing no support for Black owned businesses. Outside people or organizations come in and deploy programming that do not match community needs, resulting in apathy and frustration. One person from Community 4 explained:

*“We get tired of doing stuff because somebody else from the outside has an idea, but then we go and then the community doesn't show up and then everybody says, ‘Well, the community didn't show up.’ Well, that's because that's not what the community really wanted. So, I think it's kind of just getting to know and meet people…finding out what do the people want to build a relationship.”*

### 3.5 Lack of time and knowledge about how to connect

A number of participants explained that many people are “just trying to survive,” working multiple jobs, raising children, and/or caring for older family members. Others emphasized the need to increase awareness of existing community grassroots efforts to reduce duplication, collaborate, and act together. One resident of Community 2 explained how their family commitment limits the extent to which they can be involved:

*“I am a server. I have two younger children. They have an older sibling that still lives with us. He has a son, my grand baby. And the mother lives with us because when she got pregnant her mother put her out. I took her in…and I'm less involved in my community because I have so much going on in my home.”*

Participants discussed challenges in connecting to resources from two different perspectives. First, they explained that people in need do not know how to access existing supports, resources, and programming. In addition, people shared the need to create linkages between existing community-based efforts to enhance collective impact. Several conversations highlighted that people need help connecting with and navigating to available services. This stems from a lack of awareness that resources exist, as well as people not knowing how to access available resources or navigate the process. Some participants knew of resources that others did not, so the open mic discussions were facilitative for individuals to make connections. This illustrated the challenges that many residents have in finding and connecting to available resources.

However, some noted that there is a disconnect between what the community needs and available services and resources. Some attributed the disconnect to a lack of effective communication and the need to share information with people on their terms and “*in a way that speaks to them*.” One person from Community 3 explained how this stems from a lack of attention to community needs:

*“What tends to happen is I'm going take this one tiny thing over here. And if I don't have to expend too much energy, but I'm going to help these poor people over here…because I want to feel good … I'm not sure it's ever communicating what we need as a community because we are so separate.”*

There was also significant conversation around the challenges of working in silos, duplication of efforts, lack of communication among community members, and lack of connectivity. Participants talked about how working in silos promotes the lack of unity in their communities and how it inhibits collective action. They explained that people often get inspired to work on a local problem but often work on their own and do not seek out others doing similar work.

### 3.6 Lack of trust in public sectors

People mentioned lack of trust in organizations, hospitals, and state agencies, noting that they make decisions without the community’s best interest and do not represent community voices. Discussion focused on a history of negative experiences, community needs not being met, and lack of communication. Others emphasized that the priorities of public sector agencies are driven by politics, ignoring community input. One person from Community 2 shared their perspective on this:

*“They do not communicate with each other. They do not take the time to expose people outside their [organization] to what they are doing. They have had a very selfish mindset, ‘This is what we do’…No, this is what you supposed to do for the community. They do not take the time to give public information.”*

### 3.7 Trust is the essential building block

The importance of trust and relationship building was common across all communities. Building trust was explained as making genuine connections, taking the time to listen, and seeing things from someone else’s perspective. It takes acknowledging the past and how the community may have been mistreated. Listening to the community and asking for feedback can help identify community assets to build upon. A participant from Community 2 explained:

*“I can say, my community is better than when I moved there about five or six years ago due to leadership in the community, people coming together and by me giving my ideas and input. The key…it's that the people that live in that community need to find a way to come together and talk to one another…all it takes is effort.”*

Participants agreed that trusted individuals are usually from within the community and trust is built through consistency, commitment, honesty, and follow through. Residents want people who come into their community to have a reliable presence and establish long-term connections. Someone from Community 2 explained:

*“A lot of organizations have come out. But I noticed…some of them were lazy. They will just be standing there, talking to the people that they came with. It was a great thing…they were feeding, and clothing, and serving the people, but everybody that's in that organization, they were pretty much just engaging with one another. You have to branch out when you go in communities and start talking to somebody you don't know. Because that's the only way that you're going to get people engaged…they didn't engage in that, so you can't expect for the people in the community to engage.” “I think it's more about follow through. When we come together and we brainstorm, when the idea has a fire for a little while and then drops off. And to me, that's where the distrust is. It's like, well you know everybody parachutes in with their program, they say they're going to do X, Y, and Z, and then they might stay for a little while and then you don't see them no more. So, it's showing up to sustain. I think it's better to do something small and sustain it than one big to-do and not do it anymore.”*

Trust develops when people listen to the community and work with them to get what they need and address problems important to them. The value of being open-minded, humble, and open to other people’s ideas was also emphasized. Multiple communication strategies are needed to reach community residents, as described by someone from Community 1:

*“We got to go to the barber and beauty shops. We just can’t rely on Facebook and Instagram, which is what seems to be popular now with communication. We’ve got to look at different ways of communicating so that the people can get the message.”*

Timing and location of outreach activities also matter. People may want to participate in an event, but because it occurs at a time that does not work for their schedule. A participant from Community 4 shared how community enthusiasm may consequently “*slide by the wayside*.” Others suggested working through trusted organizations including churches, as they are often a gathering place. Additional trusted organizations mentioned included schools, food banks, fraternities/sororities, and organizations with CHWs. Neighborhood associations were also mentioned several times.

## 4 Discussion

The purpose of our project was to identify ways to promote meaningful community engagement and equitable decision-making for sustainable community health improvement. Our team worked in partnership with communities to gather and record their thoughts and ideas on community engagement. The findings from our study underscore the relevance of the Aligning Systems for Health framework’s emphasis on elevating community voice and attending to power and equity dynamics (16). For example, participants in this study emphasized that trust is essential to building meaningful community engagement and provide tangible strategies for building trust, which is also well documented in the literature (33, 34). Our community open mic session data also highlight barriers to community engagement: a history of trauma rooted in racism and dishonesty, lack of opportunities for community voices in local decision making, and how significant social concerns can overshadow efforts to promote community engagement.

This study also provides insight into how to translate community voice into capacity building opportunities, which is acknowledged as a key for sustainable improvements in public health and health equity (35, 36). For example, in Spring 2024, CCHA (in partnership with the CHWRs) used these data to develop the Prioritizing Long-lasting Actionable Community Engagement and Equity (P.L.A.C.E.) Academy. The P.L.A.C.E. Academy aims to equip various interestholders (including community and organizational leaders, researchers, and funders) with strategies and practical tools for meaningful community engagement. Based on findings from the open mic sessions, the research team created a P.L.A.C.E. Academy curriculum that covers four key topic areas: (1) Meaningful Community Engagement, (2) Trust Building, (3) How to Effectively Engage the Community, and (4) Equity in Action. For example, community participants shared the importance of their social connections and community pride. This translated into a focus in the P.L.A.C.E. Academy on the importance of building on key community assets, rather than focusing on deficits when engaging communities. Community participants also emphasized the importance of acknowledging local history and context, as well as addressing issues that are important to them (such as social challenges), rather than focusing on predetermined outsider informed areas of focus. The P.L.A.C.E. Academy incorporated this information by sharing effective strategies to engage communities especially individuals and communities most impacted by inequities. Open mic participants frequently emphasized the critical and foundational role that trust building has; therefore, the P.L.A.C.E. Academy intentionally equipped workshop participants with tools and strategies to establish deep understanding and elevate community voices. Finally, open mic session participants requested more opportunities to be engaged in decision making to ensure new policies and programs address local needs; this informed the Academy’s broad impetus to continue building capacity for engagement among community leaders, researchers, and funders.

The pilot P.L.A.C.E Academy was conducted over 2 day long sessions with 11 individuals representing all four regions of the state. Pilot participants included CHWs, as well as other individuals who play integral roles in and are actively engaged in community-engaged work. Collectively, these participants possess extensive, firsthand knowledge of grassroots-level community conditions and a deep understanding of the needs and challenges involved in bridging gaps between decision-makers and those implementing community-based work. Drawing on their professional roles and lived experience in community-engaged work, participants were intentionally engaged in curriculum vetting to determine whether the academy’s content was appropriately designed to bridge gaps for the intended target audience. Participants also provided in-depth feedback on content and delivery, which was used to refine and strengthen the P.L.A.C.E Academy curriculum. Participants will be acknowledged as contributors to the development of the academy. P.L.A.C.E. Academy pilot participants also provided detailed feedback on the curriculum via an evaluation survey and brief interviews. Overall, the feedback was positive, but suggestions for improvements were shared, including ideas for content enhancement, accessibility and inclusion, delivery and structure, audience engagement, and connectivity. Key enhancements include the addition of more real-world case studies, hands-on exercises, and opportunities for peer discussion and networking. Other enhancements include ensuring inclusivity and accessibility with clear visuals, improved pacing, and more frequent breaks to support participant engagement and learning.

Moving forward, the team plans to pilot the revised P.L.A.C.E. Academy with the target audience for the curriculum: organizational and institutional leaders. The team also plans to seek additional funding to expand the program to different regions of the state and nationally. These co-creation opportunities are a growing area for the promotion of sustainable community engagement (37).

Consideration of these findings is also needed from the philanthropic community. Funding that allows the community, rather than the funder or the researcher, to set the agenda is needed. More time for planning and trust building is required, with longer commitments to work with communities rather than a typical short funding cycle. These investments will build community trust and capacity, improving program sustainability and community health (38). It is important to understand the community from a historical perspective, as well as how organizations are perceived, and work with community leaders and gatekeepers to acknowledge and address any barriers to participation early in the engagement process. Programs that are developed and led by CHWs (such as the P.L.A.C.E. Academy) are a promising approach to advancing connections between communities, enhancing communications, and ultimately improving health outcomes (39).

The limitations of our study should be acknowledged. While we present rich, detailed data from a broad number of community open mic sessions, our findings cannot be generalized. We acknowledge that the participants in open mic sessions were primarily female and African American, so these results may not inform efforts with other populations. Moreover, although our methodology yielded a comprehensive data set from a range of community voices, our findings are subject to the interpretation of our research team. To enhance the rigor of our findings, we implemented measures including multiple team members participating in the analysis and interpretation of results, as well as member checking with community members.

Engaging people with lived experience or who are directly impacted by policies and programs requires humility, thoughtfulness, and a shift in the usual way of conducting academic research or delivering health and social programming. A key strength of this work is a research team that includes CHWs and academic researchers, which has been previously documented as a successful research approach (21, 40). In this study, CHWs had a key role throughout the research process. With training and support from traditional research team members, CHWs had an integral role in informing the project design, participant recruitment, data collection, analysis, and dissemination. This approach builds on the local knowledge and expertise of CHWs and their sense of identification with participating communities and therefore, has the potential to yield higher quality. Future studies will be needed to continue to understand the settings and delineate the ways in which CHWs can optimize the community engaged research process.

## Data Availability

The datasets presented in this article are not readily available because the nature of the data does not allow for anonymization. Requests to access the datasets should be directed to workmanl@mailbox.sc.edu.

## Footnotes

1 In our original research plan, we called these sessions ‘picnic table’ discussions to reflect our intent to have informal community conversations. However, some community partners pointed out the racial connotations to the word, ‘picnic’. Thus, we called them ‘open mic community conversations’, a name offered by local community partners.
